# Impact of the COVID-19 Pandemic on road traffic collision injury patterns and severity in Al-Ain City, United Arab Emirates

**DOI:** 10.1186/s13017-021-00401-z

**Published:** 2021-11-19

**Authors:** Yasin J. Yasin, David O. Alao, Michal Grivna, Fikri M. Abu-Zidan

**Affiliations:** 1 Institute of Public Health, College of Medicine and Health Sciences, UAE University, Al-Ain, United Arab Emirates; 2 Department of Environmental Health and Behavioral Sciences, School of Public Health, College of Health Sciences, Mekelle University, Mekelle, Ethiopia; 3 Department of Internal Medicine, College of Medicine and Health Sciences, UAE University, Al-Ain, United Arab Emirates; 4 Emergency Department, Tawam Hospital, Al-Ain, United Arab Emirates; 5 Department of Public Health and Preventive Medicine, Second Faculty of Medicine, Charles University, Prague, Czech Republic; 6 Department of Surgery, College of Medicine and Health Sciences, UAE University, Al-Ain, United Arab Emirates

**Keywords:** COVID-19, Road traffic collision, Road safety, Injury, Death, United Arab Emirates

## Abstract

**Background:**

The COVID-19 Pandemic lockdowns restricted human and traffic mobility impacting the patterns and severity of road traffic collisions (RTCs)**.** We aimed to study the effects of the COVID-19 Pandemic on incidence, patterns, severity of the injury, and outcomes of hospitalized RTCs trauma patients in Al-Ain City, United Arab Emirates.

**Methods:**

We compared the data of two cohorts of patients which were collected over two periods; the Pandemic period (28 March 2020 to 27 March 2021) and the pre-pandemic period (28 March 2019 to 27 March 2020). All RTCs trauma patients who were hospitalized in the two major trauma centers (Al-Ain and Tawam Hospitals) of Al-Ain City were studied.

**Results:**

Overall, the incidence of hospitalized RTC trauma patients significantly reduced by 33.5% during the Pandemic compared with the pre-pandemic period. The mechanism of injury was significantly different between the two periods (*p* < 0.0001, Fisher’s Exact test). MVCs were less during the Pandemic (60.5% compared with 72%), while motorcycle injuries were more (23.3% compared with 11.2%). The mortality of hospitalized RTC patients was significantly higher during the Pandemic (4.4% compared with 2.3%, *p* = 0.045, Fisher’s Exact test). Logistic regression showed that the significant factors that predicted mortality were the low GCS (*p* < 0.0001), admission to the ICU (*p* < 0.0001), and the high ISS (*p* = 0.045). COVID-19 Pandemic had a very strong trend (*p* = 0.058) for increased mortality.

**Conclusions:**

Our study has shown that the numbers of hospitalized RTC trauma patients reduced by 33.5% during the COVID-19 Pandemic compared with the pre-pandemic period in our setting. This was attributed to the reduced motor vehicle, pedestrian and bicycle injuries while motorcycle injuries increased. Mortality was significantly higher during the Pandemic, which was attributed to increased ISS and reduced GCS.

## Introduction

Road traffic collision **(**RTC) is a major global burden with 1.35 million deaths and 50 million non-fatal injuries annually [1]. In the United Arab Emirates (UAE), RTC is the seventh cause of death and fifth cause of disability-adjusted life years (DALYs), causing approximately 8.9 deaths and 626 DALYs per 100 000 population, which is higher than other high-income countries [2, 3]. RTCs cause 47% of trauma deaths in the Abu Dhabi Emirate [4]. Al-Ain City which is located in Abu Dhabi Emirate has an advanced trauma system providing trauma care for more than 750 000 residents [5–7].

Currently, the world is under the major impact of the COVID-19 Pandemic [8]. The first case of COVID-19 was confirmed in the UAE on 29 January 2020 [9]. In response to this Pandemic, the UAE government has implemented a series of measures since February 2020 [9], including lockdown, closure of schools, staying-at-home, avoiding public gatherings, and working from home [9], which was enforced by law using severe penalties for violations [10, 11]. Accordingly, these measures are expected to affect road mobility, transport, traffic congestion, and RTCs in the UAE. It is important to study the effects of the COVID-19 Pandemic on the RTCs patterns of injury and outcomes to plan future responses to similar pandemics. We aimed to study the effects of the COVID-19 Pandemic on incidence, patterns of injury, injury severity, and outcomes of hospitalized RTCs trauma patients in Al-Ain City, United Arab Emirates.

## Patients and methods

### Ethical consideration

Abu Dhabi Health Research and Technology Ethics Committee, The Department of Health, Abu Dhabi Emirate, gave ethical approval for this study (Ref: DOH/CVDC/2021/650).

### Study design

A comparative retrospective analysis of prospectively collected data of two cohorts of patients. The first cohort was the hospitalized RTC trauma patients of Al-Ain City for the year before the COVID-19 Pandemic who died or were followed up to discharge from the hospital, and the second cohort was the hospitalized RTC trauma patients of Al-Ain City in the first year of the COVID-19 Pandemic who died or were followed up to discharge from the hospital.

### Setting

Al-Ain City is the second-largest city in Abu Dhabi Emirate, UAE. It has an estimated population of 766,009 [7]. The city has two major hospitals that received trauma emergencies prior to the COVID-19 Pandemic (Al-Ain and Tawam Hospitals). Following the outbreak of the COVID-19 Pandemic, Al-Ain Hospital was designated on 28 March 2020 as the COVID-19 hospital, and it stopped receiving trauma patients. Tawam hospital was designated a non-COVID hospital and was the only trauma receiving hospital during the Pandemic.

### Patients

All RTC trauma patients who died in the hospital or who were admitted at both Al-Ain and Tawam hospitals from 28 March 2019 to 27 March 2020 (pre-pandemic period) and those who died in the hospital or who were admitted to Tawam hospital from 28 March 2020 to 27 March 2021 (Pandemic period) were studied. During the Pandemic period, all trauma patients that presented to Tawam Hospital (the non-COVID-19 hospital) were screened by a reverse transcriptase-PCR COVID-19 test on arrival to the Emergency Department. They would be continuously managed in the Emergency Department till the PCR result comes back. They would be admitted to Tawam Hospital only if the PCR result was negative which would take around 4 h. If the test was positive, they would be directly transferred to Al-Ain Hospital (the COVID-19 hospital) for further care. If a trauma patient needs an urgent life-saving or limb saving surgery, then this procedure will be performed in Tawam Hospital under strict personnel protective equipment and disinfection precautions without waiting for the PCR result. The patient will wait in the operating recovery room till the PCR result is received to decide whether to admit the patient to Tawam Hospital or transfer him/her to Al-Ain Hospital.

### Data collection

Data were retrieved from the Abu Dhabi Trauma Registry. This is the National Registry of Abu Dhabi Emirate, which started in 2013 and collects data from 8 hospitals from Abu-Dhabi Emirate. The Abu Dhabi Trauma Registry is based on the National Trauma Database of the American College of Surgeons. Data are collected prospectively, coded, and entered by full-time trained registry nurses.

### Studied variables

Studied variables in both periods (pre-COVID period and the Pandemic period) included demography, mechanisms of injury, physiological and anatomical severity markers, ISS, hospital and ICU admission, length of stay, and death.

### Statistical analysis

Data are presented as number (percentage) for categorical data, median (range) for ordinal data, and mean (standard deviation) for continuous data. Fisher’s Exact test was used to compare the categorical data of two independent groups, while Mann–Whitney U test was used to compare continuous or ordinal data of two independent groups when performing a univariate analysis.

Trauma mortality depends on numerous predicting factors. Finding statistical significance in a single independent predicting factor in a univariate analysis is not enough to indicate that this factor is a predictor of mortality. It can be simply a confounder of another factor. Multivariate logistic regression models will address this concern if we want to know whether the Pandemic actually increased the mortality of hospitalized RTC trauma patients [12]. Accordingly, factors that had a loose *p* value of less than 0.1 in the univariate comparison between those who died and those who survived were entered into a backward logistic regression model. Statistical Package for the Social Sciences (IBM-SPSS version 26, Chicago, Il) was used for all analyses. A *p* value of less than 0.05 was accepted as significant.

## Results

**Fig. 1:**
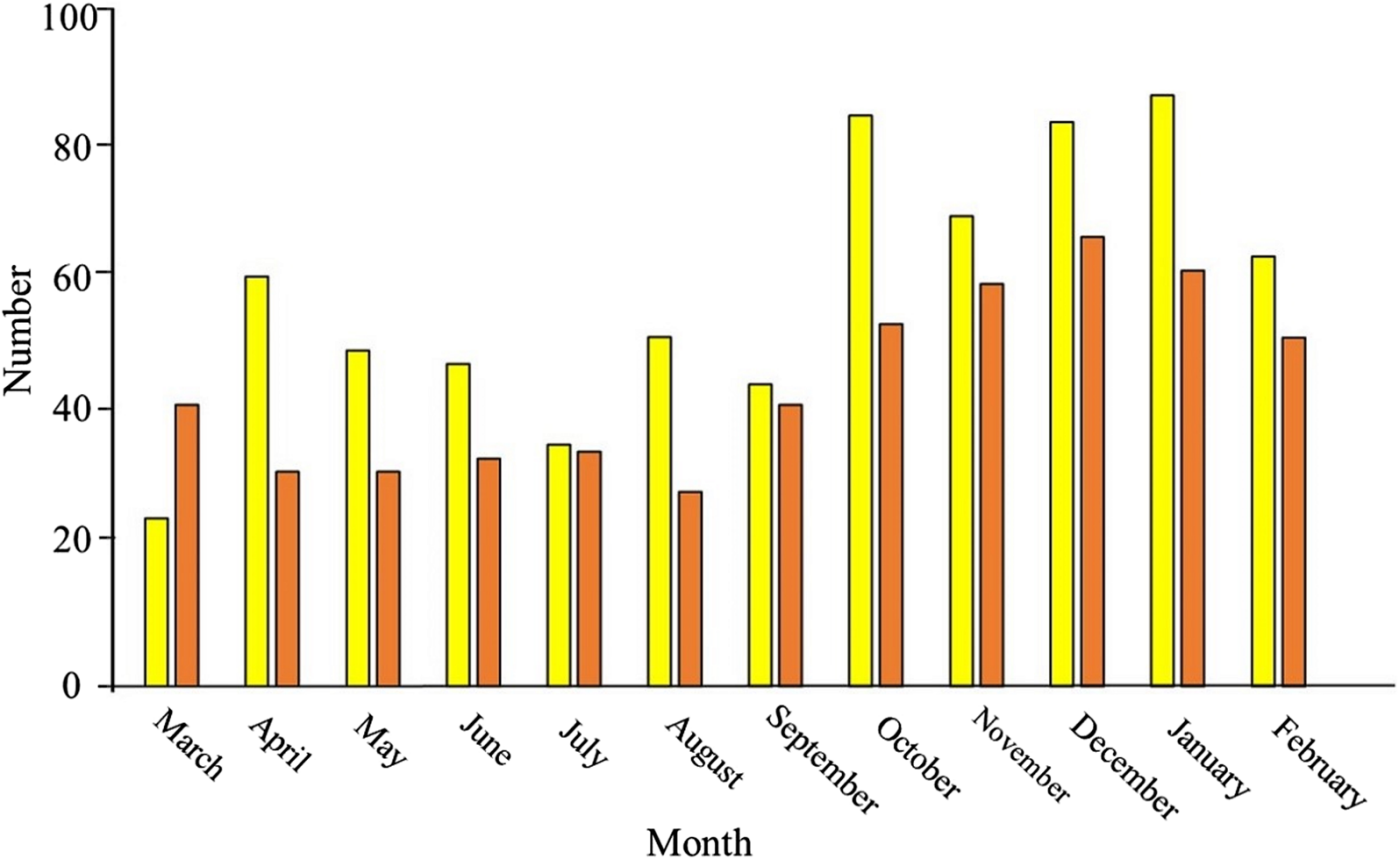
Monthly number of hospitalized road traffic collision trauma patients during the periods of March 2019–February 2020 (yellow bars) and March 2020–February 2021(red bars), Al-Ain City, United Arab Emirates

There were 750 hospitalized RTC trauma patients in the year before the COVID-19 Pandemic and 499 hospitalized RTC trauma patients during the first year of the Pandemic. This gives an annual incidence of RTC hospitalization of 97.9 /100 000 population in the year before the COVID-19 Pandemic and 65.1/100 000 population during the first year of the Pandemic. There was a 33.5% reduction in the annual RTC hospitalization in Al-Ain City. Figure 1 shows that the maximum drop was in the first five months (April-August 2020) when there was a lockdown with severe restriction of outdoor movements. Only 3 RTC patients were COVID-19 positive during the study period. They had non-threatening limb fractures and soft tissue injuries. They were transferred to Al-Ain Hospital (the COVID-19 hospital) for further care, all survived and were discharged home.

Table 1 shows the demography of the two periods. UAE nationals were significantly less during the COVID-19 Pandemic (*p* = 0.004). The mechanism of injury was significantly different between the two periods (*p* < 0.0001). Motor vehicle collisions were less during the Pandemic (60.5% compared with 72%), while motorcycle injuries were more (23.3% compared with 11.2%). RTC patients who were admitted during the Pandemic had significantly lower systolic blood pressure (*p* < 0.0001) and significantly higher respiratory rate on arrival to the hospital (*p* < 0.0001) compared with those admitted in the pre-COVID period. The mortality of hospitalized RTC patients was significantly higher during the Pandemic (4.4% compared with 2.3%, *p* = 0.045) (Table 2).

**Table 1:** Demography of hospitalized patients involved with road traffic collisions during the periods of March 2019–February 2020 (n = 750) and March 2020–February 2021(n = 499), Al-Ain City, United Arab Emirates

Variable	Pre-COVID period n = 750	COVID period n = 499	*p* value
Age	29.8 (16.1)	29.31 (14.9)	0.94
Male	616 (82.1)	413 (82.8)	0.82
Nationality			0.004
United Arab Emirates	373 (51)	205 (42.4)	
Non-UAE	359 (49)	278 (57.6)	
Mechanism of injury			< 0.0001
Motor vehicle collision	540 (72)	302 (60.5)	
Motorcycle	84 (11.2)	116 (23.3)	
Bicycle	42 (5.6)	35 (7)	
Pedestrian	84 (11.2)	46 (9.2)	
Mode of arrival			0.29
Ground ambulance	610 (81.7)	421 (84.4)	
Private car/walking-in	120 (16.1)	72 (14.4)	
Helicopter ambulance	17 (2.2)	6 (1.2)	

Data are presented as mean (SD) for continuous data and number (%) for categorical data

*p* value = Mann–Whitney U test or Fisher’s Exact test as appropriate

**Table 2:** Severity markers of hospitalized patients involved with road traffic collision during the periods of March 2019–February 2020 (n = 750) and March 2020–February 2021 (n = 499), Al Ain City, United Arab Emirates

Variable	Pre-COVID Period n = 750	COVID Period n = 499	*p* value
Systolic blood pressure (mmHg)	132.2 (23.7)	127 (19.2)	< 0.0001
Respiratory rate/minute	19.7 (4.4)	20.16 (3)	< 0.0001
Pulse (beat per minute)	93.3 (21.4)	95.01 (20.6)	0.16
GCS	15 (3–15)	15 (3–15)	0.63
ISS	5 (1–75)	5 (1–38)	0.14
ICU admission	103 (13.7)	66 (13.2)	0.87
ICU stay (days)	1.31 (4.92)	1.05 (4.17)	0.69
Hospital stay (day)	6.59 (11.9)	5.01 (7.6)	0.90
Dead	17 (2.3)	22 (4.4)	0.045

*GCS* Glasgow Coma Scale, *ISS* Injury Severity Score, *ICU* Intensive Care Unit

Data are presented as mean (SD) for continuous data, median (range) for ordinal data and number (%) for categorical data

*p* value = Mann–Whitney U test or Fisher’s Exact test as appropriate

The mechanism of injury was significantly different between those who died and those who survived (*p* = 0.03). Pedestrian injuries were more in those who died (23.1% compared with 10%), while bicycle injuries were less in them (0% compared with 6.4%) (Table 3). Those who died had significantly lower systolic blood pressure (*p* = 0.003), respiratory rate (*p* = 0.02), and GCS (*p* < 0.0001) on arrival to the hospital compared with those who survived. They had significantly higher ISS (*p* < 0.0001) and were significantly more admitted to the ICU (*p* = 0.03) compared with those who survived (Table 4).

**Table 3:** Demography of hospitalized patients involved with road traffic collision during the periods of March 2019–February 2021 in those who survived (n = 1210) and those who died (n = 39), Al-Ain City, United Arab Emirates

Variable	Alive n = 1210	Dead n = 39	*p* value
Age	29.7 (5.7)	27.6 (14.7)	0.56
Male	996 (82.3)	33 (84.6)	0.83
Nationality			0.87
United Arab Emirates	562 (47.6)	16 (45.7)	
Non-UAE	618 (52.4)	19 (54.3)	
Mechanism of injury			0.03
Motor vehicle collision	816 (67.4)	26 (66.7)	
Motorcycle	196 (16.2)	4 (10.3)	
Bicycle	77 (6.4)	0 (0)	
Pedestrian	121 (10)	9 (23.1)	
Mode of arrival			0.06
Ground ambulance	996 (82.5)	35 (89.7)	
Private car/walking-in	190 (15.7)	2 (5.1)	
Helicopter ambulance	21 (1.7)	2 (5.1)	
Period			0.045
Pre-COVID	733 (60.6)	17 (43.6)	
COVID	477 (39.4)	22 (56.4)	

Data are presented as mean (SD) for continuous data and number (%) for categorical data

*p* value = Mann–Whitney U test or Fisher’s Exact test as appropriate

**Table 4:** Severity markers of hospitalized patients involved with road traffic collision during the periods of March 2019-February 2021 in those who survived (n = 1210) and those who died (n = 39), Al-Ain City, United Arab Emirates

Variable	Alive n = 1210	Dead n = 39	*p* value
Systolic blood pressure (mmHg)	130.9 (20.1)	85.26 (63.5)	0.003
Respiratory rate/minute	20 (3.6)	14.1 (12.6)	0.02
Pulse (beat per minute)	94.28 (19.1)	78.17 (65.8)	0.99
GCS	15 (3–15)	3 (3–14)	< 0.0001
ISS	5 (1–45)	25 (9–75)	< 0.0001
ICU admission	159 (13.1)	10 (25.6)	0.03
ICU stay (days)	1.16 (4.6)	2.54 (5.4)	0.02

*GCS* Glasgow Coma Scale, *ISS* Injury Severity Score, *ICU* Intensive Care Unit

Data are presented as mean (SD) for continuous data, median (range) for ordinal data and number (%) for categorical data

*p* value = Mann–Whitney U test or Fisher’s Exact test as appropriate

Table 5 shows the outcome of the logistic regression model that predicts mortality in our studied patients. The model was highly significant with a high R squared (*p* < 0.0001, Nagelkerke R2 = 0.83). The R square indicates that 83% of the variation of the data can be explained by the selected factors. The significant factors that predicted mortality were the low GCS (*p* < 0.0001), admission to the ICU (*p* < 0.0001), and the high ISS (*p* = 0.045). COVID-19 Pandemic had a very strong trend (*p* = 0.058) for increased mortality.

**Table 5:** Backward logistic regression model defining significant factors affecting mortality

	Coefficient	S.E	Wald	*p* value	OR	OR 95% CI	
						Lower	Upper
GCS	− 0.82	0.14	35.54	< 0.0001	0.44	0.34	0.58
ICU admission	− 4.46	1.22	13.36	< 0.0001	0.01	0	0.13
ISS	0.08	0.04	4.00	0.045	1.08	1	1.16
Pandemic period	1.48	0.78	3.60	0.058	4.37	0.95	20.05
Constant	3.94	1.65	5.74	0.017	51.42		

*SE* standard error, *OR* odds ratio, *CI* confidence interval

## Discussion

Our study has shown that the COVID-19 lockdown measures have reduced the annual incidence of RTC hospitalization by 33.5%. The mortality of hospitalized RTC patients doubled during the Pandemic. Both the absolute number and relative percentage of motorcycle injuries increased during the Pandemic compared with motor vehicles collisions which had reduced. Although the absolute numbers of bicycle and pedestrian injuries decreased, the relative percentages stayed almost the same compared with other road users.

The reported reduction of the incidence of RTCs varied in different studies. Although some studies reported a reduction in the incidence [13–15], others found that it did not change [16, 17]. Similarly, the incidence of bicycle injuries in other studies varied depending on whether the use of bicycles was encouraged as the preferred mode of exercise/transport during the Pandemic [15, 18–21].

There were fewer motorcyclists, bicyclists, and pedestrian road users in the UAE compared with 4-wheel vehicles users before the COVID-19 Pandemic [22], which is reproduced in the current study. Commercial food delivery was allowed during the Pandemic, which was mainly on motorcycles explaining the increased motorcycle injuries. The changes in the mechanism of injury during the Pandemic are related to the restrictions on vehicle mobility [23–25]. Furthermore, there was a significant decrease in the hospitalized UAE nationals. Majority of UAE nationals work as governmental officers who were asked to stay at home during the lockdown. In contrast, majority of non-UAE nationals work as manual laborer in essential daily services and were asked to continue their work as usual. Police reports from other UAE Emirates support our current study. Road traffic collisions reduced in Sharjah (by 84%) [26], Dubai (by 46%) [27], and Ajman (by 45%) [28]. Numerous similar studies worldwide demonstrated a reduction in the incidence of RTCs and hospitalization rate during the COVID-19 Pandemic restrictions [23, 24, 29–32], with a reducing effect in the number of RTCs presentations in trauma centers worldwide [31].

The mortality of hospitalized RTC trauma patients doubled during the Pandemic. This is most probably related to health care difference between the two periods. We anticipate that the delay of the admission in the COVID-19 period (around 4 h) for severely injured patients when waiting for the PCR results especially to the ICU had a negative effect on the patients’ outcome. This showed a strong trend in the logistic regression model (*p* = 0.058) which did not reach statistical significance possibly due to the small sample size (Type I statistical error).

Systolic blood pressure and respiratory rate were significantly worse during the Pandemic in our study, although ISS and GCS were unchanged. Other authors reported significant changes both in the anatomical and physiological markers of severity between the two periods [14, 15, 33]. Our current study has shown that low GCS, higher ISS, and admission to ICU are the most significant clinical predictors of mortality among patients who died, similar to our reported previous studies before the pandemics [34–36].

### Limitation of the study

We acknowledge that our study has certain limitations. *First,* the reduction of RTC patients during the COVID-19 Pandemic may not be solely due to COVID-19 lockdown measures but as a continuation of the decreasing trend of RTCs in our city over the last decade [36]. *Second,* our study is from a single city in UAE and may not represent the whole UAE. *Third,* our study did not include RTC trauma patients treated in the emergency department who were discharged home nor those having minor injuries who did not seek medical advice and those who died on the scene. *Fourth*, our study was short of proving that the Pandemic increased mortality of RTCs despite the strong trend due to the relatively small sample size. *Finally*, the detailed mechanism of the RTCs and environmental conditions, like the speed of the vehicles, were not available.

## Conclusions

Our study has shown that hospitalized RTC trauma patients were reduced by 33.5% during the COVID-19 Pandemic compared with the pre-pandemic period in our setting. This was attributed to the reduced motor vehicle, pedestrian and bicycle injuries while the incidence of motorcycle injuries increased. Mortality doubled during the Pandemic, which was attributed to increased ISS and reduced GCS. There was a very strong trend for the Pandemic to increase the mortality.

## Data Availability

There are no additional data available to share with the readers. Data can be shared with the Editor of the Journal if requested.

## Abbreviations

CI: Confidence interval
GCS: Glasgow coma scale
ICU: Intensive care unit
ISS: Injury severity score
MVC: Motor vehicle collision
OR: Odds ratio
RTC: Road traffic collision
SD: Standard deviation
SE: Standard error
UAE: United Arab Emirates
UK: United Kingdom
USA: United States of America
